# Effect of transcutaneous electro-stimulation in postoperative rehabilitation pain treatment in thoracic surgery: a randomized clinical trial

**DOI:** 10.1186/s13063-024-08613-9

**Published:** 2024-12-19

**Authors:** Daniel David Álamo-Arce, Daniel López-Fernández, Raquel Medina-Ramírez, Martín Vílchez-Barrera, Pilar Etopa-Bitata, Maria del Pino Quintana-Montesdeoca, Aníbal Báez-Suárez, Jorge L. Freixinet

**Affiliations:** 1 Physical Therapy, University of Las Palmas de Gran Canaria, Las Palmas, Spain; 2 University of Las Palmas de Gran Canaria, Las Palmas, Spain

**Keywords:** Postoperative pain, Electrotherapy, Thoracic surgery, Physical therapy modalities

## Abstract

**Background:**

Chest pain is one of the most difficult problems to solve after thoracic surgery. Its correct control is often quite difficult, which can cause complications due to an ineffective cough and superficial respiratory movements.

**Methods:**

This study has been designed with the purpose of studying the value of transcutaneous electrical stimulation (TENS) in the postoperative pain rehabilitation of thoracotomy. A prospective and randomized study has been developed. The patients (*n* = 109) have been treated after hospital discharge with physiotherapy for 3 weeks. Three groups have been established: experimental (*n* = 37), control (*n* = 35), and placebo (*n* = 37), experimental and placebo including the application of TENS during the physiotherapy protocol. Postoperative pain (McGill test) and spirometry have been studied before and after treatment.

**Results:**

The largest between-group discrepancy occurred between the experimental and control groups, 16.77 points (*p* < 0.001). Spirometry has shown an improvement in FVC (27.11%) and FEV1 (28.68%) (*p* < 0.001) in the experimental group, which was statistically significant compared to the other groups.

**Conclusion:**

The use of TENS, as an adjunctive treatment to physiotherapy, leads to an improvement in pain control and spirometry values in patients after thoracic surgery, without producing side effects with the technique. These findings provide physiological evidence for the use of TENS in post-pulmonary surgery and may form the basis for the development of pain managed-based programs in clinics and hospitals.

**Trial registration:**

NCT04964973 (ClinicalTrials.gov). First registration: July 16, 2021.

Protocol: https://clinicaltrials.gov/study/NCT04964973.

**Supplementary Information:**

The online version contains supplementary material available at 10.1186/s13063-024-08613-9.

## Background

Chest pain is one of the most difficult problems to solve after thoracic surgery [1, 2]. Its correct control is often quite difficult, which can cause complications due to an ineffective cough and superficial respiratory movements. Apart from that, the pain could also provoke secretion retention, lung atelectasis, and even pneumonia [3, 4]. In addition, insufficient treatment of postoperative pain also causes a slower recovery of mobility, delaying the incorporation to daily life activities [5].

Transcutaneous electrical stimulation (TENS) is a technique that attempts to establish pain control by applying electrical current through superficial electrodes [6]. It is originally based on the control gate theory of Melzack and Wall [7]. In addition, recent studies have shown TENS can also produce analgesic effects through the generation of endogenous opioids in the central nervous system [8], and TENS also appears to have an effect on descending inhibitory activity, relayed via the midbrain periaqueductal gray (PAG) and the rostral ventral medulla (RVM) in the brainstem, which has anti-nociceptive effects [9]. Moreover, TENS has been used in various surgical procedures [10–13]. Its use may reduce the need for analgesics and, therefore, minimize side effects [14]; however, its use as a quick ambulatory pain tool together with respiratory exercises after a pulmonary surgery was not well studied. The aim of this study has been designed with the purpose of studying its value as an adjunct to postoperative outpatient quick rehabilitative treatment of thoracotomy. In this sense, the research questions which have been proposed in this study are the following ones:

- Is transcutaneous electrical nerve stimulation effective for the pain during the rehabilitation approach after thoracic surgery?
- Are there pain relief and spirometry changes related to pulmonary function after the application of transcutaneous electrical nerve stimulation in postoperative rehabilitation of thoracic surgical patients?

## Methods

### Participants

This is a randomized clinical trial with double blind. Its objective is to evaluate the analgesic effectiveness of TENS in thoracic surgical patients. It has been authorized by the Hospital’s Research Ethics Committee (CEIC 15012) and has been carried out in the period between June 2021 and March 2022. All patients have signed the informed consent. Patients aged 18 to 80 years, which were affected by pulmonary or mediastinal pathology and which required thoracic surgery, were included. The types of surgeries used were posterolateral thoracotomy, anterolateral thoracotomy, and axillary thoracotomy. The different surgery patients with pacemakers, diseases with chronic need for analgesic drugs, and history of drug addiction, those who required readmission after surgery, those lost to follow-up, and those who have not signed the informed consent were excluded from the analysis. This study was conducted in the respiratory rehabilitation unit in a general university hospital from Spain.

### Measures

The study has been developed, in all cases, using the following techniques:

1. Preoperative level: physical stimulation of abdominal-diaphragmatic breathing and techniques to improve thoracic expansion, through muscle training and cardiovascular exercises [15–17]
2. Postoperative level: on the 7th postoperative day, the patient has already been discharged from hospital. All patients have had the same analgesic treatment. A McGill test has been performed to analyze the level of immediate postoperative pain [18]. McGill test (pain rating index) (PRI) measured three dimensions: sensory, affective, and evaluative. It consisted of 66 words (pain descriptors) [19]. A score from 0 (no pain) to 66 (severe pain) has been assigned. A simple spirometry has also been performed using the spirometer Datospir Micro Sibelmed. The values of FVC (forced vital capacity) and FEV1 (forced expiratory volume in one second) are shown in percentages according to the theoretical values [3]

There were different techniques to improve postsurgical scars and their flexibility and to recover respiratory parameters based on the American Thoracic Society recommendation (endurance training 20 to 40 min per session, strength training using resistance training dumbbells, and respiratory physical therapy using breathing retraining, cough techniques, and mucus clearance, applied three times per week for 30 min’) with an improvement in pain control (depending on the study group) [20].

### Design and procedures

According to the proposed randomization, the sample has been randomly divided into three groups (a collaborator did the randomization, through a software):

1. Control group. Patients have performed the conventional postsurgical program without adding TENS
2. Experimental group. The application of TENS has been added to the physical therapy program. It had a frequency of 100 Hz and a phase duration of 100 μs for a period of 30 min (through channel 1 of the TENS equipment), receiving and feeling the patient the physical sensation of the current
3. Placebo group. In this group, the same program as group 2 was proposed, using, in this case, channel 2, which did not activate the electric current, and the patient did not receive any physical sensation

**Fig. 1:**
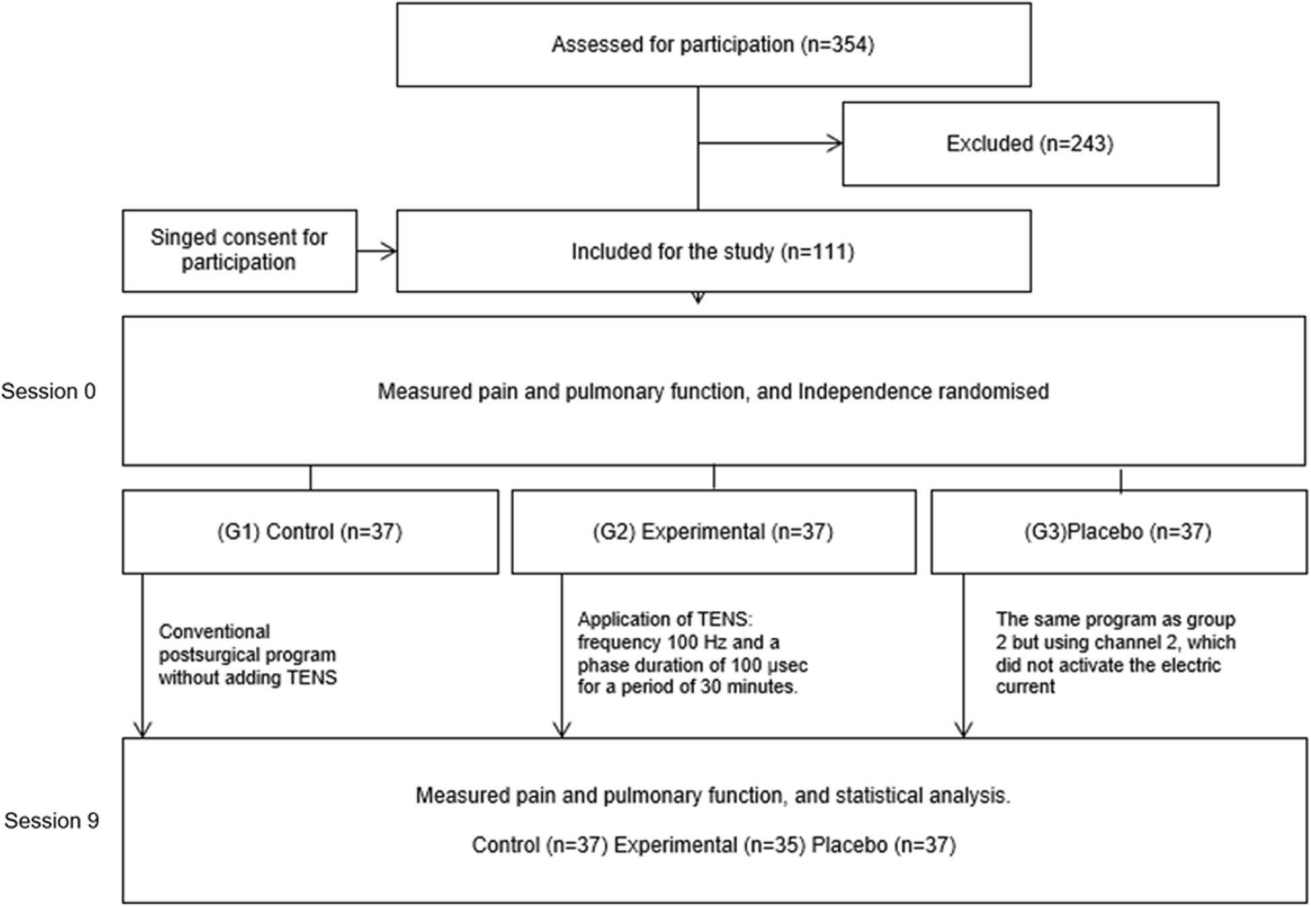
Diagram of the intervention process developed

In the experimental and placebo groups, after cleaning the skin with a soap solution, two adhesive electrodes were placed on the proximal and distal part of the scar, in order to stimulate the intercostal nerve upward. See more information in Fig. 1.

After treatment, PRI and spirometry were carried out, with identical measurements to those performed before treatment. The differences obtained in these parameters, before and after treatment, have been measured.

### Statistical analysis

#### Calculation of the sample size

The sample size was calculated with the G * Power 3.1 tool in order to develop a comparative study to detect possible differences in the numerical variable that assesses pain, in the average scores obtained in each of the 3 treatment groups (control, experimental, and placebo). A level of statistical significance (*α*) of 5%, a statistical power (1 − *β*) of 80%, and an effect size equal to 0.3 have been considered, resulting in an allocation of 37 individuals per group, with 111 cases in total.

We used the IBM SPSS version 27.0 for the statistical analysis. The categorical variables have been summarized using percentages and absolute frequencies. The non-parametric binomial and chi-square tests have been used to contrast the equality of the proportions of the categories. The numerical variables have been summarized using the mean and standard deviation. To contrast their normality, the Kolmogorov–Smirnov and Shapiro–Wilk tests have been used. In addition, Student’s *t* test of paired samples or the nonparametric Wilcoxon test was analyzed for comparison of means of two related samples. In the case of the comparison of means of three independent samples, the one-way ANOVA procedure or the non-parametric Kruskal–Wallis test were used. The level of statistical significance considered has been *p* < 0.05.

## Results

The three treatment groups presented homogeneous characteristics in terms of diagnosis and type of intervention. The diagnoses included were bronchogenic carcinoma (control group *n* = 27; experimental group *n* = 31; placebo group *n* = 22) and undiagnosed pulmonary nodule (control group *n* = 10; experimental group *n* = 4; placebo group *n* = 15). The kinds of surgical intervention were pulmonary lobectomy (control group *n* = 27; experimental group *n* = 31; placebo group *n* = 22) and pulmonary segmentectomy (control group *n* = 10; experimental group *n* = 4; placebo group *n* = 15). There have been no significant differences among the groups.

The 243 participants excluded had to assist with chemotherapy or radiotherapy treatment. Two patients did not receive their allocated intervention: two in the experimental group due to development of other treatment recruitment during the protocol, for chemotherapy treatment.

### Clinical outcomes

The initial selected sample comprised a total of 111 patients, excluding two cases due to loss to follow-up, resulting in a total of 109 cases, predominantly male (*n* = 65; 59.6%).

A global improvement in PRI and spirometric values was found in all parameters at the end of the study period, with statistically significant differences in experimental group. PRI post-surgery was 27.83 (10.99), and at the final stage of the treatment, it was 21.14 (10.68), with a mean difference of 6.64 (7.98). FVC post-surgery was 56.64 (12.37)%, and at the final stage of the treatment, it was 70.61 (15.49)%, with a mean difference of 13.97 (13.15). FEV1 post-surgery was 58.98 (12.26)%, and at the final stage of the treatment, it was 73.10 (15.50)%, with a mean difference of 14.12 (13.99).

**Fig. 2:**
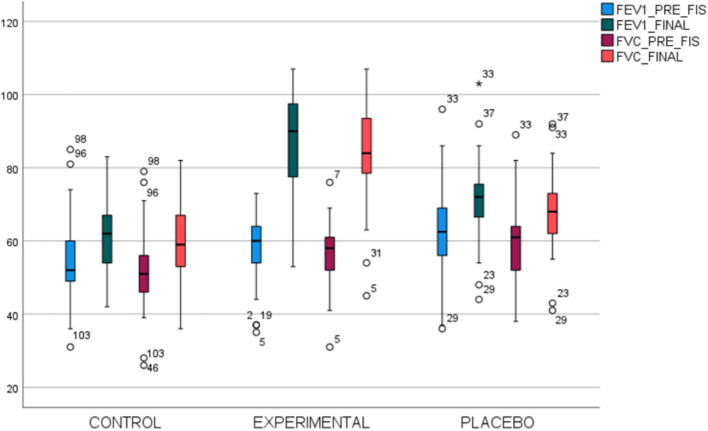
Box diagram representing the results comparing the three groups, for the variables pre-physical therapy and final FEV1 and FVC pre and final physical therapy. The term “PRE_FIS” means before the physical therapy program

If we compared the three groups, a decrease in PRI and an improvement in FVC and FEV1 could be observed at the end of the treatment. It has been statistically significant in the three values, favorable for the experimental group (Table 1) (Fig. 2).

**Table 1:** The characteristics of the groups studied are described

Variables		CONTROL (*n* = 37)	EXPERIMENTAL (*n* = 35)	PLACEBO (*n* = 37)	*p*-value
Gender	Men	32.3% (21)	38.5% (25)	29.2% (19)	0.201
	Women	36.4% (16)	22.7% (10)	40.9% (18)	
Age		66.27 (10.55)	64.86 (12.14)	65.62 (10.93)	0.867
PRI Total	Initial (Post Qx)	30.59 (8.96)	25.71 (12.42)	26.92 (11.12)	0.143
	Final	28.35 (7.61)*^♣^	11.69 (7.53)*	22.86 (9.33)*^♣^	<0.001
FVC	Initial (Post Qx)	51,81 (11.14)^♣^	56.66 (8.54)	59.83 (11.14)^♣^	0.005
	Final	59.68 (9.72)*	83.77 (14.04)*	69.11 (12.07)*	<0.001
FEV1	Initial (Post Qx)	55.00 (11.12)^♣^	58.37 (9.72)	63.54 (14.14)^♣^	<0.01
	Final	61.54 (9.56)*^♦^	87.06 (13.41)*	71.46 (11.40)*^♦^	<0.001

*p < 0.001; ^♦^*p* < 0.01; ^♣^*p* < 0.05

The values of the parameters studied before and after postoperative rehabilitative treatment are summarized in Table 2.

**Table 2:** Comparison through the treatment groups of the variables considered in the study (FEV1, forced expiratory volume in 1 s; FVC, forced vital capacity; PRI, pain rating index)

Variables		Control	Experimental	Placebo
		(*n* = 37)	(*n* = 35)	(*n* = 37)
**Gender**	Men	32.3% (21)	38.5% (25)	29.2% (19)
	Women	36.4% (16)	22.7% (10)	40.9% (18)
**Age**		66.27 (10.55)c	64.86 (12.14)	65.62 (10.93)
**PRI total**	Initial (post Qx)	30.59 (8.96)	25.71 (12.42)	26.92 (11.12)
	Final	28.35 (7.61)*§	11.69 (7.53)*	22.86 (9.33)*
**FVC**	Initial (post Qx)	51.81 (11.14)	56.66 (8.54)	59.83 (11.14)
	Final	59.68 (9.72)*	83.77 (14.04)*	69.11 (12.07)*
**FEV1**	Initial (post Qx)	55.00 (11.12)	58.37 (9.72)	63.54 (14.14)
	Final	61.54 (9.56) *	87.06 (13.41)*	71.46(11.40)*

^*^p < 0.001; ¨*p* < 0.01; ^§^*p* < 0.05

**Fig. 3:**
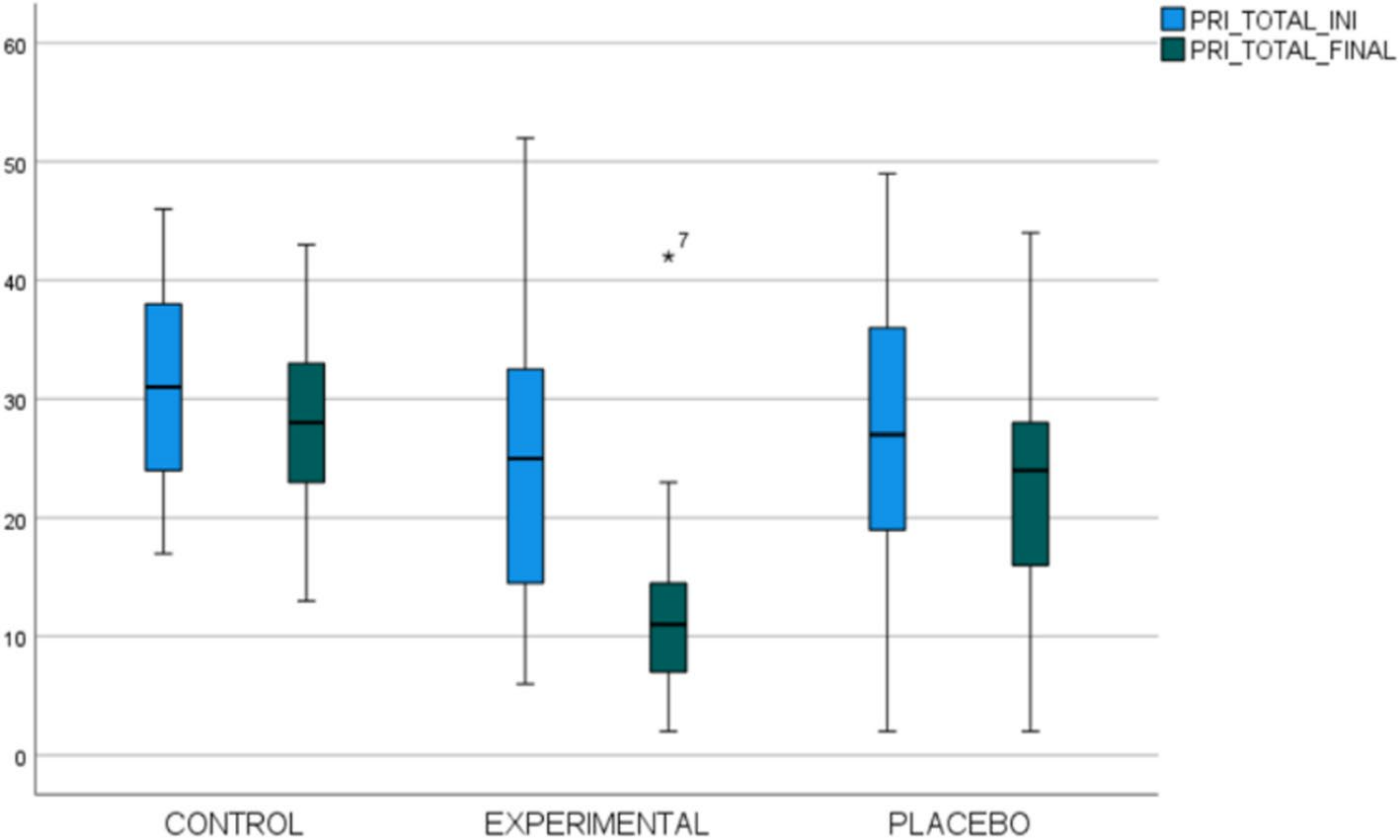
Results compared by groups of the initial and final total PRI in a box plot

The groups were compared and showed significant differences for the PRI values (Fig. 3).

The experimental group presented a great decrease in pain, followed by the placebo group and the control group. There is a great discrepancy between the experimental and the control group, 11.79 points (*p* < 0.001), followed by a difference of 9.98 points (*p* < 0.001) for the experimental group and the placebo group. The placebo group has obtained a decrease of 1.81 points (*p* = 0.033), compared to the control group.

With regard to the values recorded in spirometry, statistically significant results were also expressed. In the case of FVC, it showed 19.25% for the experimental group compared to the control group and 18.77% for the placebo group. For FEV1, the differences have been 22.14% with the control group and 19.19% with the placebo group (Table 3).

**Table 3:** Comparison of the differences among groups for the values after surgical intervention (post Qx) and at the end of the treatment (FEV1, forced expiratory volume in 1 s; FVC, forced vital capacity; PRI, pain rating index)

Group	Variables		Mean (DT)	Mean difference (DT)
Control	PRI total	Initial (post surgery)	30.59 (8.96)	− 2.24 (4.37)
		Final	28.35 (7.61)	
	FVC	Initial (post surgery)	51.81 (11.14)	7.86 (6.65)
		Final	59.68 (9.72)	
	FEV1	Initial (post surgery)	55.00 (11.12)	6.54 (5.59)
		Final	61.54 (9.56)	
Experimental	PRI total	Initial (post surgery)	25.71 (12.42)	− 14.03 (8.71)
		Final	11.69 (7.53)	
	FVC	Initial (post surgery)	56.66 (8.54)	27.11 (14.07)
		Final	83.77 (14.04)	
	FEV1	Initial (post surgery)	58.37 (9.72)	28.68 (14.05)
		Final	87.06 (13.41)	
Placebo	PRI total	Initial (post surgery)	26.92 (11.12)	− 4.05 (4.50)
		Final	22.86 (9.33)	
	FVC	Initial (post surgery)	59.83 (11.14)	8.34 (4.86)
		Final	68.17 (10.78)	
	FEV1	Initial (post surgery)	63.54 (14.14)	7.92 (8.07)
		Final	71.46 (11.40)	

## Discussion

Recovery after thoracic surgery involves achieving an improvement in respiratory function as well as minimizing postoperative pain. Therefore, postoperative physical therapy plays an important role. The main objective of this study was to assess whether a better early functional recovery is achieved by combining conventional treatment with the administration of TENS. Nowadays, this analgesic technique has been extensively studied in the immediate postoperative period, with mixed results [21, 22]. In a randomized study after posterolateral thoracotomy, little benefit was found using this technique in the initial postoperative period (Husch) [4]. Our study, with a similar design, but performed in a different moment in the postoperative evolution, has achieved better results.

Treatment with TENS can reduce the side effects derived from post-surgical pain medication [14, 23, 24]. We are not aware that its use as a complement to the classical treatment with physical therapy in the recovery of the patient, once discharged from hospital, has been subjected to a study such as this study we present. The variables used in this study have been both pain assessment by PRI, which is easy to interpret, and simple spirometry; both tests give objective values that have been widely used in previous studies [25–28]. The strengths of this study include the randomization into comparable groups, having a placebo group, and testing before and after treatment with physical therapy and TENS. A uniform treatment has been developed with the same rehabilitation protocol and identical analgesic treatment.

According to various authors, the application of TENS can generate different types of analgesia through the blockade of μ-opioid receptors or through the blockade of δ-opioid receptors. The μ-opioid receptors are generated with low frequencies (2–10 Hz), whereas the δ-opioid receptors are generated with high frequencies (50–100 Hz) [22]. Other authors have also indicated the frequency of 80 Hz and 250 μs, as optimal parameters for stimulation [29]. In our study, we have used a frequency of 100 Hz and 100 μs [9, 25, 30–32], because it is the frequency that raises the mechanical threshold [33].

With the use of TENS, a significant decrease in pain during walking and deep breathing has been described as well as an increase in exercise capacity when associated with drug treatment. A decrease in hyperalgesia has also been reported [27]. In this study, we have attempted to make use of these effects, which may be responsible for the positive results obtained. The fact that TENS is an easy-to-apply treatment makes its combination with physical therapy, from a practical point of view, very simple. Our study has shown an improvement in postoperative pain in all groups, because the intensity of pain decreases as the time elapsed since surgery increases. As in previous research, there have been no side effects. Our results, as an added value, have shown important differences between the control and placebo groups with respect to the experimental group and have been statistically significant in favor of the TENS group. Regarding the respiratory function, the improvement has also been significant, which confirms the data obtained with the PRI, because decreased pain allows greater chest expansion.

This study is the basis of the use of TENS in postoperative outpatient rehabilitation treatment in thoracic surgery. We believe that it should be confirmed with other studies with larger samples and multicenter studies in order to determine if TENS can be applied systematically as a complement to outpatient rehabilitative treatment.

## Conclusions

In conclusion, the use of TENS, as an adjunctive treatment, has led to an improvement in pain control and spirometry values with respiratory exercise in postoperative thoracic surgical patients, without producing side effects with the technique. Therefore, its utilization may be recommended in the early outpatient rehabilitation treatment of patients discharged from hospital after thoracic surgery.

## Supplementary Information

Supplementary Material 1.

## Data Availability

All data generated or analyzed during this study are included in this article.
